# Utilising emerging perspectives at the global and regional level to frame multisectoral nutrition governance landscape in Kenya

**DOI:** 10.1017/S1368980024000727

**Published:** 2024-03-20

**Authors:** Jacob Korir, Wilna Oldewage-Theron, Gladys Mugambi, Wanjiku N Gichohi-Wainaina

**Affiliations:** 1 Department of Nutritional Sciences, College of Human Sciences, Texas Tech University, Lubbock, TX 79409, USA; 2 Division of Health Promotion and Education, Ministry of Health, Nairobi, Kenya; 3 WorldFish, Jalan Batu Maung, 11960 Bayan Lepas, Penang, Malaysia

**Keywords:** Multisectoral nutrition governance, Multisectoral nutrition approach, Enabling determinants, Kenya

## Abstract

**Objective:**

Multisectoral nutrition governance (MNG) is a vital enabling determinant of improved nutrition outcomes. Despite this, it remains to be a complex phenomenon that lacks adequate understanding, especially in developing countries like Kenya. This narrative review aims to discuss the evolution of MNG, the current state of MNG, barriers and challenges, and based on these identify entry points for improvement within the complex governance structure in Kenya.

**Design:**

The Peer Review of Electronic Search Strategies (PRESS) and Preferred Reporting Items for Systematic Reviews and Meta-Analyses (PRISMA) guidelines were used to ensure rigorous and transparent identification of literature and interpretation.

**Setting:**

Kenya and developing countries with similar contexts.

**Participants:**

The review included forty-five documents (peer-reviewed articles and grey literature) that reported on MNG in developing countries.

**Results:**

We acknowledge that MNG is a complex and evolving determinant of better nutrition outcomes. The paper highlights challenges Kenya and other developing countries face such as inadequate leadership, inadequate coordination, insufficient capacity, inadequate monitoring and evaluation systems, and limited financial resources, among others. For Kenya in particular, there is inadequate understanding of what MNG is and how it can be effectively operationalised and tracked.

**Conclusions:**

To enhance understanding of MNG in Kenya, a country-specific assessment of MNG processes and impact outcomes using standard tools and defined metrics is vital. Such assessment will generate evidence of progress, successes, and challenges that will compel the government and stakeholders to invest more in multisectoral nutrition approaches to achieve its nutrition goals.

Kenya has seen significant economic growth and an increase in gross domestic product in the past decade which led to the country being classified as a low-middle-income country^(1)^. In addition, the period saw improvement in Human Development Index^(2)^ and Human Capital Index^(3)^. Despite these improvements in economic conditions and wellbeing, the country is on course to achieve only four out of nine global nutrition goals, namely stunting, childhood overweight, low birth weight and exclusive breast-feeding^(4)^. This shows that economic development does not necessarily guarantee improved nutrition outcomes and highlights the intricate interplay of multiple factors that influence these outcomes^(5,6)^. Moreover, several nutrition and health trends in Kenya continue to be of public health concern. In particular, its triple burden of malnutrition (co-existence of undernutrition, micronutrient deficiencies and rising overweight and obesity) is consistently observed from national data^(7–9)^.

Furthermore, geographical, socio-economic, and demographic disparities are still evident in the country. For instance, stunting is higher among children in rural areas (20 %) compared with children in urban areas (12 %) and it decreases with increasing wealth, from 28 % in the lowest wealth quintile to 9 % in the highest wealth quintile. Moreover, 22 % of children born to mothers with no education are stunted, as compared with 9 % of children born to mothers with secondary education and higher. Furthermore, wide variations in stunting across counties are evident with the highest prevalence being at 37 % and lowest at 9 %^(7,10)^. In combination, the issue of micronutrient deficiencies is still widespread, especially among children, adolescent girls, and women of reproductive age^(8)^. The most common micronutrient deficiencies are iron, folic acid, iodine, vitamin A and zinc with varying prevalence based on age and sex. Indeed, recent studies indicate that at least 50 % of children under the age of 5 years, adolescent girls and women of reproductive age are suffering from one or more micronutrient deficiencies^(8,11,12)^.

Along with nutrition outcomes related to undernutrition, obesity in adult men and women has almost tripled in the past 20 years^(9,13,14)^. In fact, the non-communicable diseases risk factor projections for Kenya by the WHO in 2019 showed a concerning trend with approximately half of adult men and women with overweight or obese^(4)^. In addition, hunger, food insecurity and poor diets continue to remain as important determinants of malnutrition^(15)^. The prevalence of undernourishment (an indicator of the extent of hunger) and severe food insecurity in Kenya is at 28·5 % and 26·1 %, respectively^(16)^.

The aforementioned nutrition situation necessitates the need for innovative and effective approaches to not only address immediate and underlying but also address enabling determinants of malnutrition. However, most efforts are currently targeted towards addressing immediate and underlying determinants of malnutrition with less emphasis on enabling determinants such as nutrition governance which are crucial in addressing malnutrition. Multisectoral nutrition governance (MNG) is crucial in addressing the complex factors contributing to malnutrition since it directly influences both immediate and underlying determinants of malnutrition such as diets, care practices, household food insecurity and access to health services among others^(17)^. Despite being a central enabling determinant for better nutrition, MNG is complex because it is dependent on the legal and institutional structures and frameworks present. It is also influenced by various frameworks aimed at improving nutrition and how these frameworks are prioritised and operationalised^(18)^. The Kenyan situation presents a unique example of the challenges that exist in MNG due to its overall national and sub-national governance structures with most functions devolved and some under the purview of the national government. In this situation, developing an appropriate enabling environment for the successful realisation of improved nutrition outcomes is a complex task.

It is crucial to unravel the current status of MNG in Kenya as it would elucidate the potential roles of various actors both at the national and sub-national level. This would in turn enable the identification of opportunities for improved MNG and therefore support sustained improvements in nutrition outcomes and perhaps improve nutrition governance at the national and sub-national level. This is particularly important when the country has made commitments aimed at improving nutrition governance such as joining the Scaling Up Nutrition (SUN) Movement, rolling out the National Food and Nutrition Security Policy (NFNSP) and Kenya National Nutrition Action Plan (KNNAP) and committing to African Union (AU) agenda 2063 among others^(19,20)^. On a global level, understanding the status of MNG would serve as a learning opportunity for other regions or countries similar to Kenya. The paper aims to discuss the evolution of MNG, the current status of MNG, barriers and challenges in Kenya, and based on these findings propose recommendations to advance MNG. The evolution of MNG is first discussed in the context of low- and middle-income countries to provide a framing for MNG. We then discuss issues specific to Kenya to identify clear entry points for improvement to enable the achievement of national nutrition goals.

## Methods

### Study design

This is a narrative review that employed aspects of the systematic review methodology to discuss MNG.

### Search strategy

We followed the Peer Review of Electronic Search Strategies (PRESS) for systematic reviews and Preferred Reporting Items for Systematic Reviews and Meta-Analyses (PRISMA) guidelines to rigorously and transparently identify appropriate literature for this review. Alignment to PRISMA and PRESS guidelines also ensured quality data extraction, analysis and synthesis, and discussions reflect on study limitations and conclusions are drawn based on the evidence presented^(21,22)^. This study focused on findings in developing countries for the framing because they have a similar context and development trajectory and they face similar governance challenges like Kenya.

The search included peer-reviewed articles and grey literature (such as progress reports, policy briefs, working papers, bulletins and conference proceedings) that reported on MNG and related topics in developing countries. Peer-reviewed articles were accessed from electronic academic research databases, including PubMed, ScienceDirect, SCOPUS, Web of Science and Google Scholar. Grey literature was drawn from Kenya government websites and reputable international organisations such as the UNICEF, World Bank Group (WBG), FAO, WHO, United States Agency for International Development (USAID) and Development Initiatives among others. Manual searching of reference lists of original articles and grey literature was also conducted.

The search terms used in electronic databases were based on key phrases related to MNG in developing countries. Keywords and phrases were combined using Boolean operators ‘AND’ to narrow the search appropriately and ‘OR’ to expand the search. The following search string was applied to specific databases: *(‘multisectoral nutrition governance’ OR ‘multisectoral nutrition programming’ OR ‘multisectoral nutrition policy and programming’ OR ‘multisectoral nutrition policy and programming’ OR ‘nutrition governance’ OR ‘multisectoral nutrition approaches’ OR ‘integrated nutrition governance’ OR ‘inter-sectoral nutrition programming’ OR ‘cross-sectoral nutrition programming’ OR ‘intersectoral nutrition collaboration’ OR ‘systems-based approach to nutrition programming’) AND (‘developing countries’ OR ‘Africa’ OR ‘Sub-Saharan Africa’ OR ‘East Africa’) AND (‘Kenya’).*

### Eligibility criteria

The first step in the study selection was the exclusion of duplicates followed by the examination of titles and abstracts obtained. Inclusion criteria entailed English-written articles and reports published between the year 2010 to 2023 to ensure that the retrieved articles reflected the current situation and evolution since 2010. The publication period was selected because it is a time when the country made progress in operationalising MNG-related structures and processes such as joining the SUN Movement, rolling out the FNSP and KNNAP and committing to AU Agenda 2063 among others^(19,20)^. Publications included peer-reviewed articles and grey literature focusing on MNG and related areas concepts fully or partially in developing countries. Within the context of this manuscript, the term *‘documents’* is used to denote all forms of grey literature. Articles and documents that focused on governance in sectors not related to nutrition were excluded. The full text of the remaining articles and grey literature was reviewed to establish eligibility, and all relevant information and data were extracted.

### Literature analysis

Basic thematic analysis was conducted after selecting and reviewing the relevant literature. The thematic analysis was guided by the objectives of the narrative review and the contents of the articles and documents. They were grouped into three descriptive themes:

1. Overall nutrition governance context and its evolution –this theme covered how MNG has evolved and the current status of MNG globally, in Africa and Kenya.
2. Progress and challenges – this theme covered progress and challenges on MNG in Kenya. It also borrowed experiences from other similar developing countries.
3. Recommendations to enhance MNG in Kenya – as part of the synthesis and evaluation across the article, this theme provided suggestions on how the understanding and operationalisation of MNG in Kenya can be enhanced.

## Results and Discussion

**Fig. 1:**
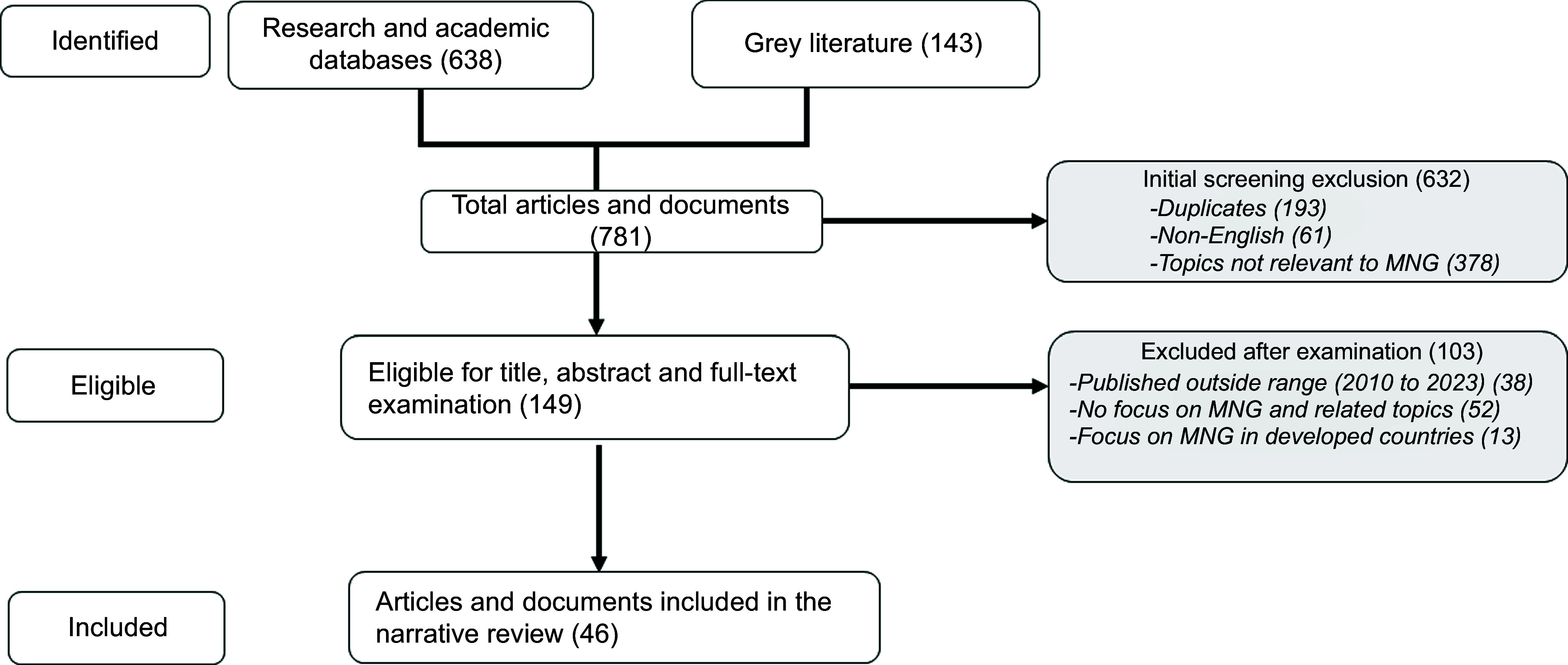
Articles and documents included and excluded during the search process. MNG, multisectoral nutrition governance

The literature search resulted in 781 articles and documents (Fig. 1). These articles and documents were reduced to 149 after 632 were excluded that were duplicates (193), not published in English (61) or had titles not focusing on MNG (378). Further screening based on year of publication (2010 to 2023), titles relevant to MNG, those focusing on developing countries and those with complete and reliable information led to the additional exclusion of 103 articles and documents. A total of 46 articles were finally assessed and synthesised in the three descriptive thematic areas.

### Origin, evolution and advancement of multisectoral approaches

#### Origin and evolution of multisectoral approaches

**Fig. 2:**
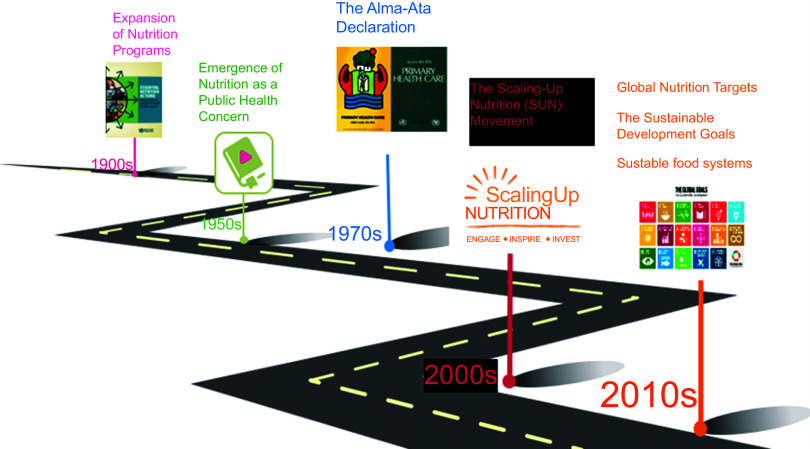
Major highlights in the historical evolution of MNG

The historical evolution of multisectoral nutrition programming and nutrition governance globally reflects a shift from the recognition of specific nutritional deficiencies to understanding malnutrition as a complex issue requiring coordinated efforts across sectors to achieve sustainable solutions^(23)^. Major highlights in the evolution of MNG include (i) The emergence of nutrition as a public health concern in the early 20th century where public health efforts primarily focused on infectious diseases. This began with nutritional deficiencies such as scurvy and rickets being identified, and interventions were initiated to address these specific deficiencies^(24)^. (ii) Expansion of nutrition programmes followed in mid-20th Century. After the Second World War, there was a growing understanding that malnutrition was not only caused by individual dietary deficiencies but also by broader socio-economic factors. National nutrition policies and programmes were established in several countries to address malnutrition comprehensively, often targeting vulnerable populations such as women and children^(25)^. (iii) The Alma-Ata Declaration adopted in 1978 at the International Conference on Primary Health Care recognised the importance of nutrition in achieving health for all (Fig. 2). It emphasised the need for integrated approaches to health, including nutrition, and called for intersectoral collaboration to address the social determinants of health^(26,27)^. (iv) The SUN Movement, launched in 2010, emerged as a global initiative to address malnutrition comprehensively. It emphasised the need for multisectoral action, bringing together stakeholders from various sectors such as health, agriculture, education and social protection. SUN countries developed national strategies and action plans, integrating nutrition into broader development agendas^(28)^. (v) The World Health Assembly (WHA) set global nutrition targets/goals, focusing on reducing stunting, wasting and micronutrient deficiencies. These targets highlighted the importance of multisectoral approaches to achieve global nutrition goals^(29)^. (vi) The UN Sustainable Development Goals (SDG) acknowledged the significance of nutrition and included a specific target (SDG 2.2) to end all forms of malnutrition by 2030. The SDG reinforced the need for multisectoral actions, recognising that nutrition is influenced by various factors beyond the health sector^(30–32)^.

At the same time, the importance of food systems and food environment has gained significant attention globally. Significant efforts include the development of the food systems conceptual framework by the High Level Panel of Experts (HLPE) on Food Security and Nutrition in 2017. The HLPE food systems framework emphasises the importance of bringing on board various actors in the food system to impact nutrition and health outcomes^(33)^. In addition, actions to increase agricultural production, transform food environments for healthy diets, mitigate climate change, productively engage the private sector and influence public policy priorities have also gained pre-eminence^(34)^. Based on this evolution, the recognition of MNG as a tool to harness the collective efforts of all actors for improved nutrition and health has become crucial.

#### What is multisectoral nutrition governance?

**Fig. 3:**
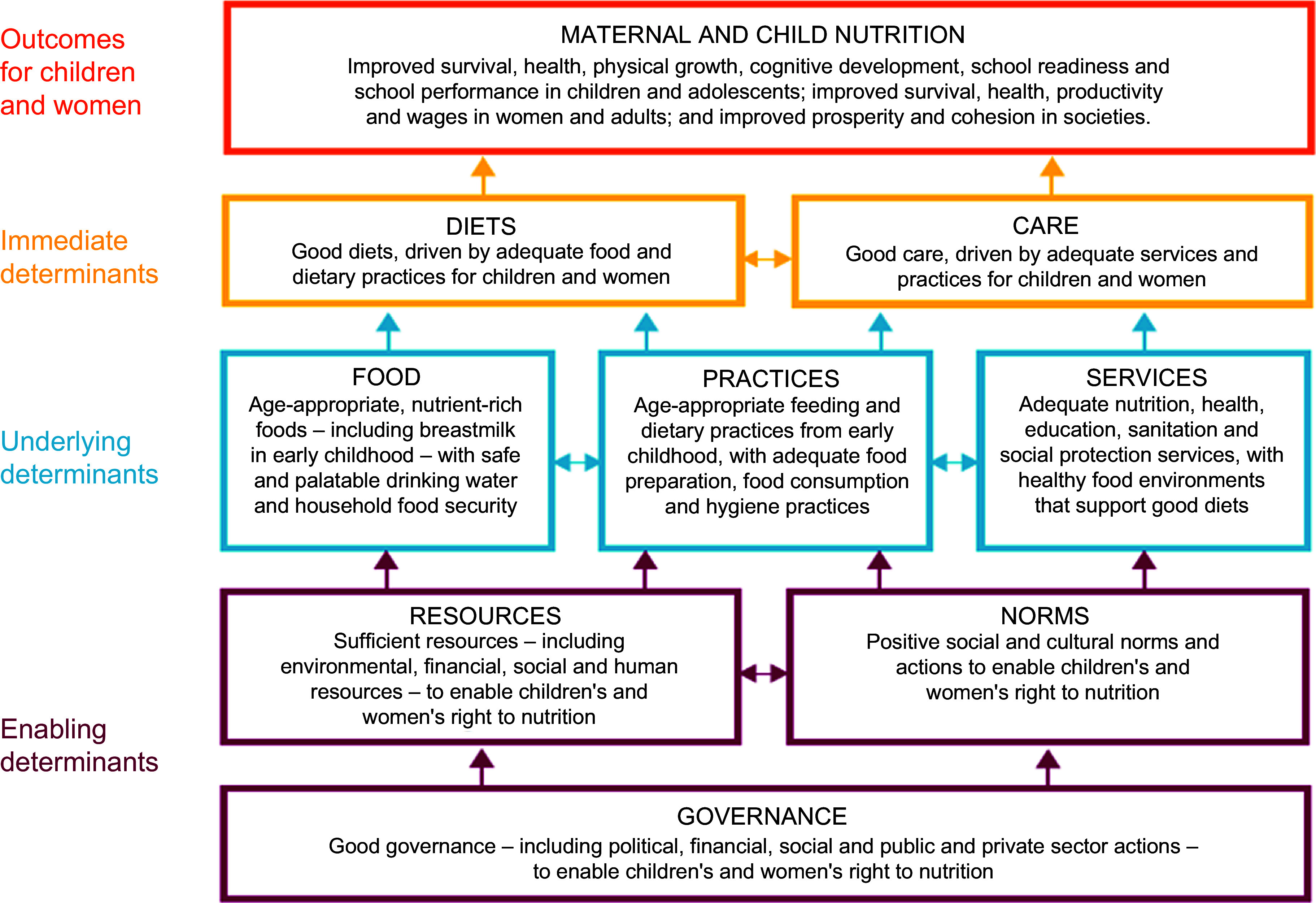
UNICEF conceptual framework for malnutrition

‘Multisectoral nutrition governance (MNG)’ is an evolving term that is often used interchangeably with ‘multisectoral nutrition programming’, ‘multisectoral approach’, ‘integrated nutrition governance’, ‘intersectoral nutrition collaboration’ and ‘systems-based approach to nutrition programming’ among others^(10,18,35–38)^. As part of this evolution, various frameworks have been developed in the past three decades in a bid to understand the complex scenario of MNG as an enabling determinant of nutrition outcomes. They include the recently revised and commonly applied UNICEF framework for malnutrition^(17)^ (Fig. 3), conceptual model of the food and nutrition system^(39)^, WHO health systems framework^(40)^, governance framework^(41)^, Lancet nutrition framework^(42)^, United Nations Systems Standing Committee on Nutrition (UNSCN) global governance for nutrition^(43)^, systems thinking and action for nutrition^(37)^, governance and leadership in agri-food systems and nutrition^(44)^, among others.

**Fig. 4:**
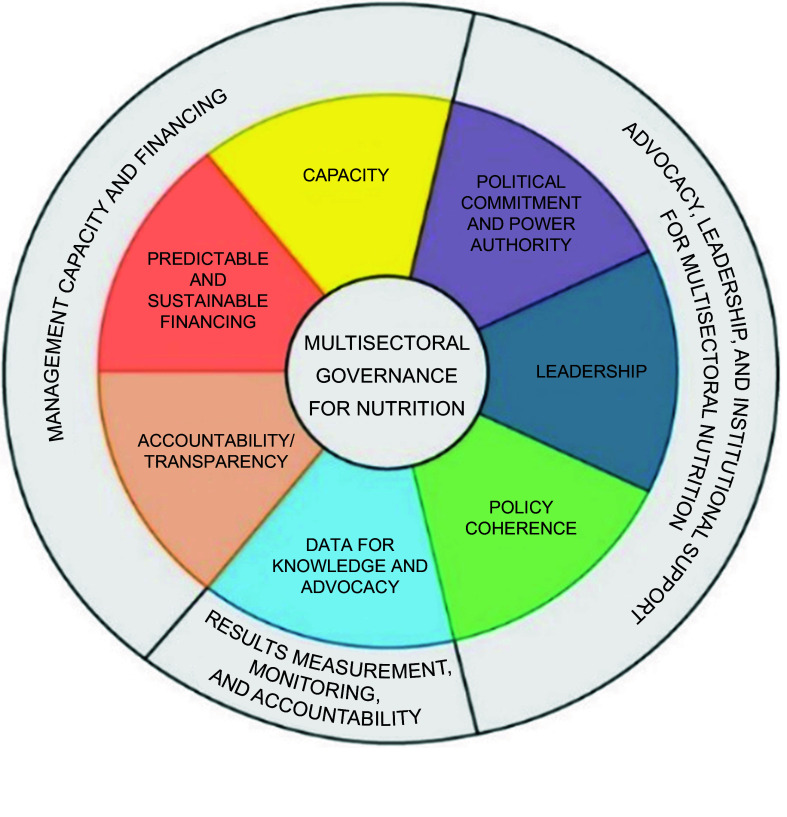
Multisectoral nutrition governance framework

The most recent is the MNG framework developed by Subandoro, Holschneider and Ruel-Bergeron (on behalf of the World Bank) (Fig. 4)^(45)^. The World Bank framework is an adaptation of governance and leadership in agri-food systems and nutrition by Gillespie, van den Bold and Hodge^(44)^. The World Bank MNG framework adds value by clearly describing themes and domains that contribute to MNG which makes it straightforward to conceptualise the concept of MNG. The themes include advocacy, leadership, institutional support, results measurement, management capacity and financing for multisectoral nutrition among others.

Considering the frameworks highlighted above, we define MNG in this paper as the iterative process of decision-making, policy development, implementation of strategies, monitoring and accountability aimed at improving nutrition outcomes across various sectors and levels. It includes political commitment and power, leadership, multisectoral coordination, policy coherence, accountability, adequate capacity, predictable and sustainable financing, results measurement, monitoring, and accountability all working in a synergistic manner and across various sectors.

#### Multisectoral nutrition governance as an enabling determinant of better nutrition outcomes

MNG is the cornerstone to addressing the immediate, underlying and enabling determinants of malnutrition and improving nutrition for four fundamental reasons. First, malnutrition is caused by a complex and interrelated set of factors, including poverty, lack of access to nutritious food, poor maternal and child health practices and weak health systems, which cannot be addressed by one sector^(18,45,46)^. Second, it is beneficial in ensuring that the causes of malnutrition are systematically addressed and resources across multiple sectors are leveraged for increased impact and sustainability in addressing malnutrition^(10,37,47)^. Third, integrated multisectoral actions can promote equitable and sustainable development in all sectors by addressing the root causes of malnutrition and reducing disparities in nutritional outcomes^(25)^. Fourth, it provides an opportunity to leverage synergies across various sectors and among existing local systems, programmes, and structures for greater impact^(37,48)^.

In recent years, there has been an increased focus on strengthening MNG structures at sub-national, national and global levels. Many governments in collaboration with various stakeholders have established high-level institutional structures, developed policies and increased efforts to coordinate and monitor multisectoral nutrition efforts, although progress is slow^(49)^. Addressing the triple burden of nutrition as well as the nutrition outcomes disparities requires evaluation of MNG efforts to understand progress, successes and challenges. This exercise would optimise MNG and lead to greater progress in sustainably addressing malnutrition.

#### Multisectoral nutrition governance in Africa

MNG in the African continent has undergone significant evolution over the years, a trend similar to the global level^(35)^. This evolution can be attributed to various factors, including growing recognition of the complex nature of malnutrition, increasing awareness of its socio-economic and health implications, and a shift towards a more holistic and integrated approach to addressing malnutrition^(23)^. This is envisioned in the AU Agenda 2063 and other frameworks^(20)^.

One notable development is the growth of ‘multisectoral nutrition approaches’ which was later called MNG. Traditionally, nutrition interventions were primarily focused on the health sector, with a narrow focus on addressing undernutrition through nutrition-specific interventions such as therapeutic feeding programmes, breast-feeding and micronutrient supplementation^(50)^. Under the agriculture sector, interventions were focused on production/availability of food as the primary goal with little focus on other food security pillars^(33)^. However, there is now a broader recognition that malnutrition is a multidimensional problem influenced by various factors, including agriculture, education, social protection, water and sanitation, and women’s empowerment. As a result, there has been a shift towards a multisectoral approach that involves collaboration and coordination among different sectors to address the enabling environment determinants of food and nutrition insecurity comprehensively^(18,51)^.

Evidence of this evolution can be found in national nutrition policy frameworks, strategies and multisectoral action plans adopted by various African countries. For example, countries like Burkina Faso, Mozambique, Senegal, Uganda and Tanzania among others have developed National Multisectoral Nutrition Action Plans (NMNAP) that outline strategies and interventions to be implemented across sectors^(25)^. These plans typically involve ministries responsible for health, agriculture, education and social welfare among others. Furthermore, these frameworks forge strong links with other continental initiatives such as AU Agenda 2063 and Malabo Declaration among others which calls for a multisectoral approach to address malnutrition in all its forms^(52)^.

In the past, nutrition governance in Africa was often fragmented, with limited coordination and weak institutional mechanisms^(53)^. However, there has been a growing recognition of the need for effective governance to drive the multisectoral nutrition agenda. To strengthen nutrition governance, many African countries have established dedicated national nutrition coordinating bodies. These bodies are responsible for coordinating and overseeing nutrition-related activities across sectors. The establishment of national SUN Movement multistakeholder platforms in several African countries, for instance, has brought together governments, civil society organisations, UN agencies, donors and other stakeholders to strengthen national nutrition governance and implementation^(28)^. Furthermore, there is an increasing emphasis on accountability and monitoring in nutrition governance. African countries are progressively investing in data collection systems, monitoring frameworks, and evaluation mechanisms to track progress and ensure transparency in nutrition programming^(45,54)^. The focus on progress assessment and accountability on MNG is helping in identifying gaps and challenges in implementation for evidence-based decision-making, although more effort is required in harmonising MNG metrics and indicators in countries like Kenya.

#### Examples of multisectoral nutrition approaches in the African context

There are instances of sector-wide attempts to enhance multisectoral programme implementation (process) and improve the nutrition situation (outcomes) across the globe. An example is the Seqota Declaration in Ethiopia which has facilitated the championing of high-level commitment, prioritisation and financing for nutrition across sectors^(55)^. On the other hand, Rwanda and Mozambique are examples of countries that have strengthened multisectoral nutrition governance at the sub-national level and have led to improvement in nutrition outcomes^(45)^. Uganda and Senegal have implemented multisectoral nutrition coordination bodies with convening powers at the national level^(56,57)^. Finally, Malawi is in the process of implementing multisectoral financial and programme indicator tracking mechanism which intends to enhance commitment and accountability for nutrition financing and results^(58)^. In summary, there are various experiences and lessons that countries in Africa can learn from the planning and operationalising of various components of MNG for improved nutrition outcomes.

#### Metrics used to assess multisectoral nutrition governance and potential associations with nutrition outcomes

One challenge that has emerged with the implementation of MNG is inadequate metrics or tools to: (i) guide how integration across actors occurs, (ii) define process success, and (iii) link MNG to improved nutrition and health outcomes. MNG is a complex and relatively recent phenomenon which makes it challenging to define appropriate metrics at the process and outcome level. To ensure comprehensive MNG assessments, the analysis should cover policy/programme design, capacity assessment, monitoring and evaluation systems, intersectoral coordination, impact evaluation, cost-effectiveness analysis, and equity assessment among others^(59–61)^. However, covering all these areas is complicated by the fact that MNG is a continuous process bringing together various stakeholders at different levels^(62)^. As a result, most attempts at standardising measures of nutrition governance have used national-level benchmarks based on available data, such as the presence or absence of certain policy documents, budgetary allocations and staffing levels which often fall short of providing a true picture of the status^(63,64)^.

The five recent and widely cited indices to assess MNG are as follows: (i) the WHO Nutrition Governance Index (WHO’s NGI) which ranks governments on their ‘commitment’ (willingness to act) and ‘capacity’ (readiness to act) to improve nutrition, (ii) the Hunger and Nutrition Commitment Index (HANCI) which ranks governments on their political commitment to addressing undernutrition while seeking to measure what governments achieve and where they fail^(65)^, (iii) the Political Commitment (for Nutrition) Rapid Assessment Tool which measures a country’s level of political commitment and identifies opportunities to advance food and nutrition on governmental agendas^(66)^, and (iv) MNG framework by World Bank which aims at qualitatively assessing enablers and barriers of MNG at the country level^(45)^. The recently developed NGI by Tufts University is the first standardised approach to quantifying the MNG in relation to national plans of action to accelerate improvements in nutrition^(64)^. In addition to these global frameworks and tools, individual countries have also developed their metrics to assess MNG. These metrics often align with global frameworks but may incorporate country-specific indicators and priorities. For instance, Ethiopia developed the National Nutrition Monitoring, Evaluation, and Research System (NNMERS) to monitor and evaluate multisectoral nutrition interventions at the national and sub-national level^(67)^. This has been replicated in other developing countries under the National Information Platforms for Nutrition (NIPN) initiative^(68)^.

It is evident that there is a lack of universally agreed tools and metrics for empirically measuring the MNG processes and outcomes. Kenya presents an excellent opportunity to apply existing tools and further refine them in line with the evolving nutrition context.

### Multisectoral nutrition governance in Kenya (evolution and barriers/challenges)

#### Kenya’s commitment to tackle malnutrition

The Constitution of Kenya (2010) guarantees the right to food and adequate nutrition and health in line with the UN Universal Declaration of Human Rights (UDHR) and other global declarations^(69)^. The right to adequate nutrition is further envisioned in the Kenya Vision 2030 and Medium-Term Plans (MTP) which are key documents that outline the government’s priorities and guide national planning and budgeting^(70)^.

Kenya’s resolve to address malnutrition has seen various policies, strategies and programmes developed and implemented. The NFNSP 2012 (and its implementation framework 2017–2022) and KNNAP 2018–2022 are the overarching frameworks guiding nutrition programming in the country. The KNNAP as the document that operationalises the NFNSP 2012 spells out the role of relevant line ministries/sectors such as health, agriculture, social protection, trade and industry, education and water, sanitation, and hygiene among others both at the national and county level^(19)^. KNNAP also provides umbrella guidance to counties which are at various levels of developing their County Nutrition Action Plans (CNAP)^(10,19,71)^. In addition, sectors such as health, agriculture, social protection, trade, education, water and sanitation among others have their sector-specific policies and plans that also intersect with the nutrition improvement agenda in Kenya^(19,71,72)^.

#### Multisectoral nutrition governance context in Kenya

**Fig. 5:**
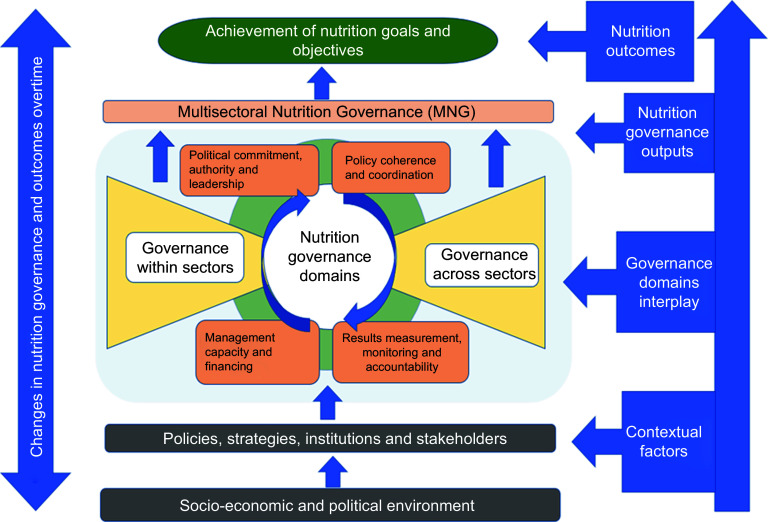
Conceptual framework for multisectoral nutrition governance in Kenya

As outlined, the achievement of MNG and nutrition goals in Kenya is a complex and multifaceted process bringing together various ministries and stakeholders at different levels (Fig. 5). Convening various stakeholders, processes and institutions to scale up nutrition in a harmonised way continues to present a challenge in Kenya. This is primarily due to the involvement of diverse sectors and stakeholders, compounded by the decentralised governance system in which some functions such as primary health care have been devolved to counties, while others such as basic and higher education are still under the oversight of the national government^(10,19)^.

At the basic level, the broader socio-economic and political context determines how the country is governed. This broader context shapes the overarching framework of nutrition policies, institutions and stakeholders, which are organised around government ministries^(73)^. An optimal interplay of MNG domains in an enabling environment where government and non-governmental stakeholders collaborate in a coordinated manner will lead to MNG and the achievement of nutrition goals and outcomes. This requires political commitment and power, leadership, multisectoral coordination, policy coherence, accountability, adequate capacity, predictable and sustainable financing, results measurement, monitoring and accountability within and across sectors. The successful interplay of MNG domains is critical to the achievement of nutrition goals and outcomes in Kenya. Effective understanding of MNG in Kenya therefore requires a multi-perspective assessment of progress, enablers and barriers and how various factors in the conceptual framework interact. Prioritising these factors will lead to effective MNG and the attainment of improved nutrition outcomes^(10,19)^.

### Challenges in achieving multisectoral nutrition governance

Several challenges that developing countries are encountering as they strive to establish effective MNG have been identified. These challenges can be attributed to various factors, including inadequate leadership and guidance, limited resources, weak institutional capacity, fragmented coordination mechanisms, and competing priorities among others^(18,25,37,45,48,64)^. Challenges specific to Kenya are given in the following sections.

#### Inadequate leadership and guidance

The absence of clear and comprehensive policies addressing multisectoral approaches to nutrition hinders the implementation and coordination of actions across sectors. Inadequate leadership and guidance to sectors also contribute to limited political will and goodwill to act especially in the context of competing demands for investments from other programmes^(10)^. For instance, the ruling party’s (Kenya Kwanza) manifesto (2022–2027) commits to eradicate malnutrition within 5 years. These ambitious targets require well-defined strategies to give a clear indication of what forms of malnutrition and what actions will be undertaken at the national and county level so that this is achieved^(74)^. In addition, coordination among key ministries relevant to addressing malnutrition is lacking. For instance, the NFNSP is domiciled in the Ministry of Agriculture Livestock and Fisheries (MOALF) which perceives nutrition as largely a food security issue, while the KNNAP is domiciled in MoH which perceives nutrition largely as a health issue, which has led to coherence challenges. Moreover, preventive services such as nutrition are given less prominence as compared with curative services in Kenya’s Universal Health Coverage (UHC) policy^(75)^. There is therefore a need to foster a coherent policy framework that provides guidance on multisectoral nutrition programming.

#### Inadequate coordination frameworks

Effective MNG requires various sectors to collaborate and coordinate coherently. Insufficient indices and mechanisms for multisectoral collaboration and coordination within and among various sectors involved in nutrition hinder the effective implementation of integrated nutrition interventions^(72,76)^. There is a need for Kenya to put emphasis on coordination frameworks that bring together relevant sectors, especially non-traditional sectors to facilitate multisectoral collaboration and coordination.

#### Insufficient institutional and technical capacity

Inadequate guidance on the integration of nutrition interventions across sectors, as well as limited capacity building initiatives, hinder effective implementation. Studies conducted in developing countries have highlighted the need for technical assistance and training to support the planning and operationalisation of multisectoral nutrition programmes^(23,30,45,77,78)^. A study to assess the enabling environment for nutrition-sensitive agriculture in Ethiopia, Kenya and Uganda established that institutional and technical capacity to plan and implement multisectoral nutrition actions remains a challenge^(79)^. There is a need for future nutrition capacity assessment and development efforts to integrate MNG.

#### Inadequate monitoring and evaluation systems for multisectoral nutrition governance

Kenya faces challenges in monitoring and evaluating MNG due to a lack of defined systems and capacity for monitoring and evaluation. Various actors have inadequate guidance to structure MNG and common tools or metrics to conduct MNG process and impact evaluation. Insufficient data collection systems and fragmented data sources make it difficult to obtain comprehensive and accurate information on nutrition interventions across sectors. The complex and multifaceted nature of nutrition programming, coupled with the presence of various determinants of malnutrition, makes it challenging to attribute changes in nutrition outcomes solely to multisectoral interventions. Capturing this complexity and ensuring comprehensive measurement of nutrition outcomes can be challenging^(10)^. Moreover, the majority of studies have focused on short-term outputs, such as changes in knowledge or behaviour. Only a few studies have evaluated the association between MNG and nutrition outcomes^(80)^. There is therefore need for Kenya to adopt and implement approaches and tools that will comprehensively capture the multidimensional nature of MNG and nutrition outcomes. These challenges highlight the need for improved data collection, strengthened monitoring and evaluation systems, and robust evaluation methods to effectively track and measure the impact of multisectoral approaches to nutrition in the country.

#### Limited financial resources

Kenya faces resource constraints that hinder the effective implementation of multisectoral nutrition programming. Resource mobilisation and commitment are yet to meet the requirements stipulated in the KNNAP. Limited resource allocation not only affects implementation but also impedes the capacity to assess progress^(76)^. Generation of evidence on MNG progress, successes, challenges and impact will bolster advocacy for additional resources for multisectoral nutrition actions in Kenya.

## Conclusion

Kenya has made significant commitments towards addressing nutrition challenges. However, sustained efforts are required to maintain progress in some areas such as reducing stunting, addressing the growing public health problem of overweight and obesity, and alleviating micronutrient deficiencies, among others. Despite being a central enabling determinant for better nutrition outcomes in the country, MNG is a complex phenomenon that lacks adequate understanding in Kenya. Enhancing the understanding of MNG progress and challenges in the Kenyan context is essential for optimising processes and systems that facilitate effective nutrition actions within and across sectors. Indeed, MNG challenges and entry points identified in this paper are generic and/or deciphered from literature that does not directly assess MNG. To enhance a clear understanding of MNG in Kenya, a detailed assessment of MNG processes, its measurement (if any) and its impact on intended outcomes using standard tools and defined metrics is vital. Such assessment will generate specific evidence that serves as a stepping stone upon which the government and stakeholders can invest more in MNG to achieve nutrition goals.
